# Laboratory Validation of Xpert Chlamydia trachomatis/Neisseria gonorrhoeae and Trichomonas vaginalis Testing as Performed by Nurses at Three Primary Health Care Facilities in South Africa

**DOI:** 10.1128/JCM.01430-17

**Published:** 2017-11-27

**Authors:** Remco P. H. Peters, Lindsey de Vos, Liteboho Maduna, Maanda Mudau, Jeffrey D. Klausner, Marleen M. Kock, Andrew Medina-Marino

**Affiliations:** a Department of Medical Microbiology, University of Pretoria, Pretoria, South Africa; b Department of Medical Microbiology, CAPHRI School for Public Health and Primary Care, University of Maastricht, Maastricht, The Netherlands; c Anova Health Institute, Johannesburg, South Africa; d Research Unit, Foundation for Professional Development, Pretoria, South Africa; e David Geffen School of Medicine, University of California—Los Angeles, Los Angeles, California, USA; f Department of Epidemiology, University of California—Los Angeles, Los Angeles, California, USA; g National Health Laboratory Services, Tshwane Academic Division, Pretoria, South Africa; Brigham and Women's Hospital

**Keywords:** antenatal care, molecular diagnostics, STI screening, human immunodeficiency virus, sexually transmitted diseases

## LETTER

The introduction of molecular diagnostic tests provides an important step to address the burden of sexually transmitted infections (STIs), especially Chlamydia trachomatis, Neisseria gonorrhoeae, and Trichomonas vaginalis. Recently developed Xpert CT/NG (for C. trachomatis/N. gonorrhoeae) and TV (for T. vaginalis) assays provide opportunities to detect these STIs in resource-limited settings (1). When performed by staff at primary health care (PHC) facilities, patients can be provided results and treatment within 2 h.

We implemented Xpert CT/NG and TV assay testing of HIV-infected pregnant women at three PHC facilities in Pretoria, South Africa (2), and conducted a laboratory validation of Xpert results obtained at these facilities.

Participants self-collected three vulvovaginal swabs. The first swab was immediately processed and tested using Xpert CT/NG and TV assays (Cepheid, Sunnyvale, CA) at the PHC facility per the manufacturer's instruction and as described elsewhere (3). The two other swabs were shipped to the Department of Medical Microbiology, University of Pretoria, for additional laboratory and molecular analysis.

For laboratory confirmation, DNA was extracted from the second swab using the High Pure PCR template preparation kit (Roche Diagnostics, Basel, Switzerland) and analyzed with the Presto^Plus^ CT/NG/TV assay (Microbiome, Ltd., Houten, The Netherlands) as per the manufacturer's instruction. The Presto^Plus^ assay has reported high concordance with the Roche Cobas CT/NG assay and the TIB Molbiol LightMix TV assay (4, 5). Specimens with discordant results between Xpert and Presto^Plus^ were confirmed with the Anyplex II STI-7 assay (Seegene, Seoul, South Korea) per the manufacturer's instruction (6).

**FIG 1:**
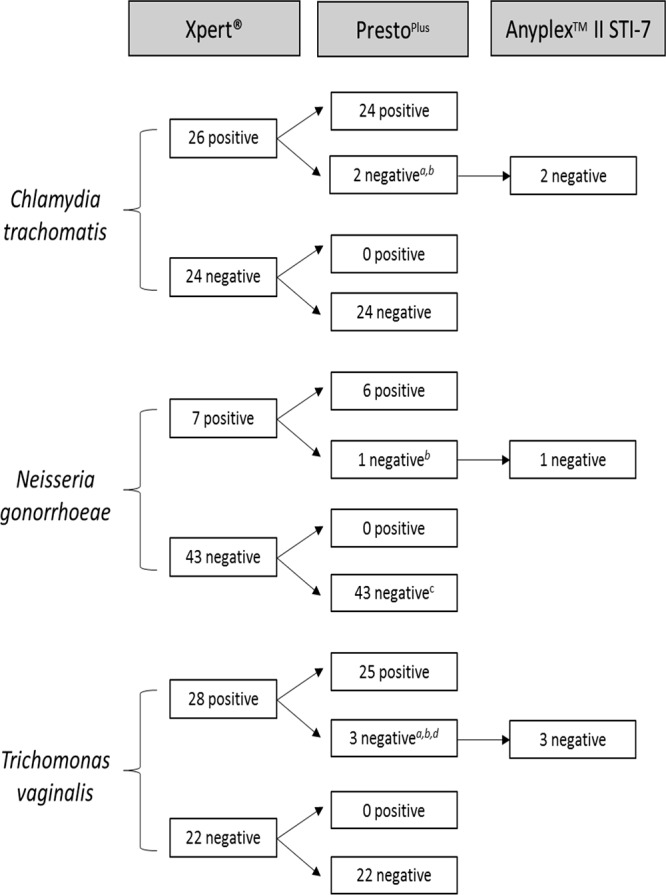
Results of laboratory validation of Xpert CT/NG and TV tests of self-collected vaginal swabs from 50 HIV-infected pregnant women. Footnote *a* indicates this patient had discordant results for both Chlamydia trachomatis (Xpert *C_T_* value of 38.3) and Trichomonas vaginalis (Xpert *C_T_* value of 39.9). Both showed amplification in the initial Presto^Plus^ test (*C_T_* values: for Chlamydia trachomatis, 38.7; for Trichomonas vaginalis, 39.4) but were negative in the Presto^Plus^ repeat test as per the manufacturer's instruction. Footnote *b* indicates this patient had discordant results for all three microorganisms, with Xpert *C_T_* values as follows: for Chlamydia trachomatis, 34; for Neisseria gonorrhoeae, 34.0 for NG1 probe and 35.2 for NG2 probe; for Trichomonas vaginalis, 37.0. Footnote *c* indicates the initial Presto^Plus^ test was low positive (*C_T_* value of 37.6), but the specimen tested negative upon Presto^Plus^ retest. Footnote *d* indicates the *C_T_* value of this specimen was 39.7 in the Xpert assay.

The results from 50 randomly selected specimens by Xpert testing identified that 26 were C. trachomatis positive, 7 were N. gonorrhoeae positive, and 28 were T. vaginalis positive. Xpert and Presto^Plus^ results were concordant for 47/50 (94%) of participants (Fig. 1). Two of the three discordant results may be attributed to sampling and testing variation as suggested by the high Xpert cycle threshold (*C_T_*) values (>38 cycles). While the initial Presto^Plus^ test also gave equivocal *C_T_* values of >38 cycles for C. trachomatis and T. vaginalis for one of these patients, repeat Presto^Plus^ and confirmatory test results were negative. In addition, three Xpert-negative specimens had initial equivocal Presto^Plus^ results but were negative upon repeat testing. This highlights the challenges with interpretation of low-positive results in molecular tests. The third patient was positive for all three STIs by Xpert and negative for all three by Presto^Plus^ and Anyplex. We attribute this discordance to either an inadvertent specimen exchange or mislabeling.

Our study is limited by the fact that confirmation by retesting was not conducted using GeneXpert assays, as additional swabs were specifically collected for nucleic acid extraction to be used for research purposes. However, we used two established molecular detection assays that have a similar range of technical performance to Xpert (1, 2, 7, 8). Repeat Xpert testing of specimens with high *C_T_* values was not performed, whereas equivocal results in Presto^Plus^ were retested as per the manufacturer's instruction.

In conclusion, we demonstrate that reliable STI diagnoses can be obtained from self-collected vaginal swabs through Xpert CT/NG and TV testing by nurses at PHC facilities in South Africa. This observation supports the feasibility of implementation of easy-to-use molecular tests for STI diagnosis in resource-constrained settings.
